# A Pilot Evaluation of a Smartphone Application for Workplace Depression

**DOI:** 10.3390/ijerph17186753

**Published:** 2020-09-16

**Authors:** Daniel A.J. Collins, Samuel B. Harvey, Isobel Lavender, Nicholas Glozier, Helen Christensen, Mark Deady

**Affiliations:** 1 Black Dog Institute, Faculty of Medicine, University of New South Wales, Randwick, NSW 2031, Australia; s.harvey@unsw.edu.au (S.B.H.); i.lavender@blackdog.org.au (I.L.); h.christensen@blackdog.org.au (H.C.); m.deady@unsw.edu.au (M.D.); 2 Brain and Mind Centre, Central Clinical School, Faculty of Medicine and Health, University of Sydney, Sydney, NSW 2050, Australia; nick.glozier@sydney.edu.au

**Keywords:** depression, smartphone, mobile app, workplace, eHealth, mHealth, mental health, prevention, anxiety

## Abstract

Interventions delivered via mobile apps show particular promise in tackling the burden of common mental disorders. Appropriately targeting these interventions to at-risk populations is critical to their success. This pilot study aimed to assess the usability, feasibility, acceptability, and preliminary effects of an app-based intervention designed to target depressive symptoms in a stressed working population. Anchored, a smartphone app including a 30-day program of mindfulness and cognitive and behavioural therapeutic components, was tested in a pre-post pilot study with participants recruited via social media advertisements. Eligible participants (N = 81) were Australian adults who were employed and reported elevated stress levels on a single-item screening measure. Follow-up assessment occurred 5 weeks after baseline. The primary outcome measure was change in depressive symptoms, with secondary outcomes measuring change in anxiety, wellbeing, stress, resilience, exercise, alcohol use, absenteeism, and work performance. User feedback and in-app data were analysed to assess engagement and intervention adherence. At follow-up, there were significant reductions in depressive symptoms (t_61_ = 6.35; *p* < 0.001) and anxiety symptoms (t_60_ = 7.35; *p* < 0.001), along with significantly reduced cases of likely new onset depression compared to baseline (24% vs. 6%, *p =* 0.012). Significant improvements were also seen in wellbeing (t_60_ = −5.64; *p* < 0.001), resilience (t_60_ = −3.89; *p* < 0.001), stress (t_61_ = 11.18; *p* < 0.001), and alcohol use (t_60_ = 3.40; *p* = 0.001). Participants reported no significant changes in work performance, absenteeism or exercise. There were satisfactory levels of app usability, feasibility, and acceptability. Most participants found the app easy to use (93.2%), understood the app content well (83.0%), and rated the app highly or very highly overall (72.9%). User feedback suggestions were predominantly focused on improving app navigation and user interface. This pilot study provides encouraging evidence that Anchored has potentially beneficial effects, and is usable, feasible, and acceptable as an app-based intervention for the working population experiencing elevated stress. Further testing of Anchored in a randomised controlled trial is required to investigate its efficacy as an intervention for workplace mental ill-health.

## 1. Introduction

Depressive disorders are now the third leading cause of disease burden worldwide, with anxiety disorders also contributing significantly to years lived with disability [1]. Given the substantial overlap between poor mental health and other disabling conditions such as neurological and musculoskeletal disorders, the true burden of mental illness has been estimated to account for up to a third of years lost to disability globally [2]. Due to the magnitude of the problem and the moderate efficacy of treatments, it is unlikely that traditional treatment alone can adequately alleviate the burden [3]. Consequently, scalable and population-wide approaches are needed to reduce incidence and impact.

Internet-delivered (eHealth) prevention programs can provide a cost-effective alternative to traditional approaches, by enabling wider dissemination and scalability of effective preventative strategies [4]. Encouragingly, a recent meta-analysis found that eHealth prevention interventions had small positive effects on symptom reduction for depression and anxiety, with similar findings for both universal and selective/indicated prevention interventions [5]. An efficient means to provide eHealth interventions is via mobile/smartphone applications (mHealth), which have the potential to be distributed widely to maximise population reach [6]. Apps enable flexible user engagement, allowing individuals to access interventions at their convenience and facilitating real-time monitoring of mood and behaviour [7]. Mobile technology also necessitates new intervention formats delivered via frequent brief exchanges. Several meta-analyses of randomised controlled trials (RCTs) involving smartphone-based mental health interventions have shown significant small-to-medium effects on anxiety and depression symptoms compared to controls, across both clinical and non-clinical populations [8,9,10].

The workplace is increasingly being recognised as a potential location for mental health intervention [11]. A meta-analysis of work-based depression prevention programs found that various types of interventions, particularly those based on cognitive behavioural models, show evidence of an ability to reduce depressive symptoms in unselected working populations [12]. However, it has been demonstrated that the most efficient means for population-level intervention is to target those at increased risk [13]. In the workplace context, stress is a key risk factor for common mental disorders such as depression and anxiety, and targeting stressed workers is a widely used approach for implementing selective interventions in the workplace population [14]. While workplace stress has been implicated as a causal factor in the development of depression and anxiety [15,16], evidence suggests that individual perception of work-related stress largely mediates the relationship with depression symptoms [17]. Therefore, a key mechanism through which individual-level interventions may improve mental health outcomes is by helping workers to manage perceived stress.

The current research team have previously piloted and evaluated through an RCT the impact of a smartphone application, HeadGear, designed to prevent depression and improve wellbeing amongst workers in high-risk, predominantly male, industries [18,19]. The app was found to be effective in the prevention of depression as well as in improving a range of mental health and work-related outcomes [20]. The present study builds on these learnings in an attempt to benefit stressed workers across industries, using a new smartphone-based intervention (Anchored). The Anchored app includes therapeutic content used successfully in previous work [20], adapted to suit a broader audience based on user feedback and expert consultation. Given its universal focus and the applicability of mHealth interventions across a range of contexts, Anchored is not intended as a workplace-specific app, although it targets workplace risk factors including work stress and relationships.

This pilot study aims to assess the usability, feasibility, acceptability, and preliminary effects of an app-based intervention designed to reduce depression and improve mental health in the general working population. The primary objective of this study is to test the impact of the Anchored app on depressive symptoms among employed Australians with elevated stress levels. This study also aims to test the impact of Anchored on anxiety, stress, wellbeing, resilience, and work productivity, and to assess participant engagement and level of satisfaction with the app.

## 2. Materials and Methods

### 2.1. Study Design

This pilot study took place from October to December 2019. Employed adults were recruited via social media to test the usability, feasibility, acceptability, and preliminary effects of the app via pre- and post-intervention questionnaires and recording of in-app data.

### 2.2. Participants

Eligible participants were Australian residents, aged over 18, currently employed, who owned a smartphone, had adequate English comprehension, and self-reported elevated stress levels. All participants provided informed consent electronically, and no identifiable information was included in the study data. The study was conducted in accordance with the Declaration of Helsinki [21].

### 2.3. Intervention/App

Anchored was developed by researchers from the Workplace Mental Health Research Team at the Black Dog Institute and University of New South Wales (UNSW, Randwick, Australia). Anchored was adapted from a previously developed app, HeadGear, originally designed for workers from male-dominated industries (MDIs) [18,19]. An RCT of HeadGear with a large sample of workers from MDIs showed that the app was effective in the prevention of both depressive symptoms and the likely onset of new depression cases, and was associated with improvements in work performance, resilience, and wellbeing [20]. For the purpose of this study, modifications to the app content and design were made through a process of consultation with users, clinical psychologists, psychiatrists, information technology professionals, and design and user experience specialists. These modifications were made primarily to ensure that Anchored had a broad appeal to workers from a range of industries. Changes included a more gender-neutral look and feel (through colour scheme, imagery, language, and theme), representation of a range of male and female “characters” in the app videos, and voiceovers using both male and female presenters. 

**Figure 1:**
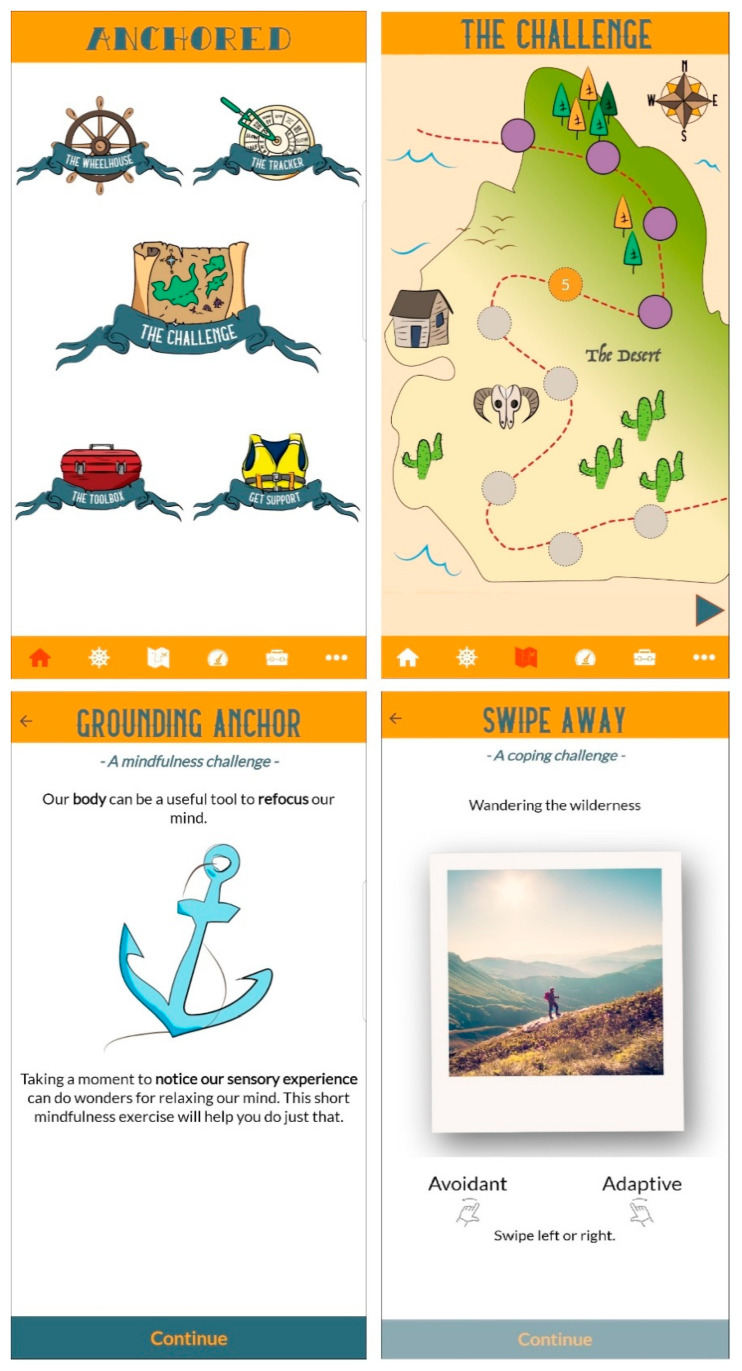
Screenshots from the Anchored app showing home screen (**top left**), challenge map (**top right**), and examples of challenge exercises (**bottom left and right**).

The main component of the Anchored app is a 30-day intervention (the “challenge”) in which users complete one task daily (5–10 min per day). The challenge features evidence-based therapeutic techniques delivered using a variety of formats including psychoeducational videos, mindfulness audio exercises, value-driven activity planning and goal-setting, and the development of coping skills (see Figure 1 for examples). In addition to the HeadGear therapeutic content (centred around behavioural activation, mindfulness, and coping skills), Anchored incorporates cognitive therapeutic elements chosen for their effectiveness in universal workplace interventions [12], along with grounding exercises and relaxation techniques designed to target stress. These additional elements were included to provide a broad range of evidence-based strategies beyond the more behaviourally focused elements preferred by workers in MDIs [18,22].

Users can also complete an optional risk calculator that assesses the risk for future common mental disorders and provides participants with personalised feedback regarding this risk [23]. Other components of the Anchored app include a tracker for monitoring mood, physical activity, and sleep, a “toolbox” of skills (which is gradually filled in as the intervention is completed), and support service helplines. The 30-day challenge and additional core features of Anchored were based on the structure of the HeadGear app, with further changes intended to improve usability and engagement, such as modified layout and addition of the physical activity and sleep tracker, based on the expressed preferences of HeadGear users [18].

### 2.4. Procedure

Participants were recruited via targeted social media advertisements calling for adults experiencing workplace stress to test a new mental health app. The advertisements stated that participants would be reimbursed for their time. Individuals who clicked on the link in the advertisements were directed to the study website which included information about study procedures and inclusion criteria. Interested individuals were then directed to read the online participant information statement and consent form on the study website.

After providing informed consent electronically, participants were required to complete a checklist to confirm that they met the inclusion criteria. They then completed a screening measure: The Single-Item Stress Question (SISQ) [24]. The SISQ is a single-item measure of current stress on a 5-point Likert scale from 1 (“Not at all”) to 5 (“Very much”) and has been validated for the screening of stress levels in a working population [25]. Those who failed to meet the inclusion criteria, or were not experiencing elevated stress, as defined by a score below 4 (“Rather much”) on the SISQ, were ineligible for the study and excluded. These individuals were provided with a list of mental health support services and were encouraged to contact the research team if they required any referral advice. Those who were eligible for the study were asked to complete the baseline questionnaire immediately after screening. Upon completion of baseline assessment, participants were provided with links to download the Anchored app to their smartphone from either the App Store (for iPhone users) or the Google Play Store (for Android phone users). Participants were required to enter their mobile number and password the first time they accessed the app, to link their in-app data and questionnaire responses. Account details were securely encrypted and were not stored with participant data.

Participants were encouraged to access Anchored daily for a period of 30 days. Participants received a text message invitation to complete online post-intervention assessment 5 weeks after completing baseline (with up to two reminder emails for non-completers).

All participants who completed both the baseline and post-intervention assessments received a $40 Visa gift card to reimburse them for their time. This study was approved by the University of New South Wales (UNSW, Randwick, Australia) Human Research Ethics Committee (HC190109).

### 2.5. Measures

The primary outcome was depressive symptoms, measured using the Patient Health Questionnaire-9 (PHQ-9) [26]. The PHQ-9 is a reliable and valid 9-item measure of depression severity over the previous two weeks and is sensitive to change [27,28]. The PHQ-9 can be used either as a diagnostic algorithm to make a probable diagnosis of major depressive disorder (MDD) or as a continuous measure with scores ranging from 0 to 27 and cutoff points of 5, 10, 15, and 20 representing mild, moderate, moderately severe, and severe levels of depressive symptoms [29].

Work-related stress was measured using the Single-item Stress Question (SISQ) [24]. As outlined above, the SISQ was administered as a screening measure; for participants included in this study the SISQ screening score constituted their baseline stress level, therefore eligible participants necessarily had a baseline score ≥4. At post-intervention, scores ranged from 1 to 5. 

Anxiety symptoms were measured using the General Anxiety Disorder-7 item (GAD-7) [30], a reliable and valid 7-item measure of generalised anxiety symptoms over the previous two weeks [31]. GAD-7 scores can range from 0 to 27, with 5, 10, and 15 representing cutoffs for mild, moderate, and severe levels of anxiety.

Participant wellbeing was measured using the 5-item WHO Wellbeing Index (WHO-5) [32]. WHO-5 scores range from 0 (worst possible quality of life) to 25 (best possible quality of life). The WHO-5 is a psychometrically sound measure with high internal consistency (Cronbach’s α = 0.84) and high levels of convergence with other measures of wellbeing.

Work performance and absenteeism were measured using items from the Health and Work Performance Questionnaire (HPQ) [33]. Work performance was assessed via the HPQ “absolute presenteeism” question (“How would you rate your overall job performance on the days you worked during the past 4 weeks?”). This question was measured on a scale from 0 to 10, with higher scores indicating higher performance (for analysis, scores were converted to a decimal ranging from 0 to 1). Short-term absenteeism was assessed via an item asking about the number of sick days in the past 28 days, with a sub-question specifying days absent for mental health reasons.

Resilience was measured using the Brief Resilience Scale (BRS), a 6-item measure designed to assess the ability to recover from stress, which has been shown to have both good internal consistency (Cronbach’s α = 0.80–0.91) and test-retest reliability [34]. Higher BRS scores indicate higher levels of resilience.

At baseline, participants were asked questions regarding demographics, mental ill-health in the prior two years, help-seeking for a mental health problem in the previous month (from any source including health professionals, telephone or online support services, family, partner or friends), whether currently taking medication for a mental health issue, and current levels of exercise and alcohol use. Help-seeking, exercise, and alcohol questions were repeated at post-intervention.

At post-intervention, app usability and engagement were measured using three questions adapted from the Mobile Application Rating Scale (MARS) [35], assessing ease of use and engagement/interest in the app design and content, along with questions regarding the usefulness of specific app features/content and the reasons for stopping app usage (if applicable). Feasibility and acceptability were assessed via questions adapted from the MARS (likelihood of recommending the app to others, and overall rating of app), as well as further questions to measure understanding of app content, and whether the app helped improve mental fitness. Participants also provided general app feedback via open response questions.

Intervention adherence was measured via collection of app usage data, which recorded the number of challenge days completed.

### 2.6. Statistical Analysis

In the HeadGear pilot study, a small-to-medium within-group effect size (Cohen’s d = 0.39) was observed on the PHQ-9 [18]. Power calculations showed that 54 participants would be required to achieve this effect size with 80% power at alpha = 0.05. To account for an expected 30% dropout rate, the intended sample size for the present study was 77.

All data were analysed using SPSS version 26. Descriptive statistics regarding participant characteristics and smartphone use data were analysed to characterise engagement and acceptability. Paired samples t-tests were used to test for differences between pre- and post-trial clinical outcomes (e.g., PHQ-9). To explore dose-response, symptom change scores were computed, and linear regression was performed to test for the effect of app engagement on symptom change. All p-values were two-sided with significance set at 5%. Effect size (Cohen’s d) was calculated using mean change/baseline SD [36]. Although this study was not targeted at a clinical population, exploring clinically significant changes can be useful in determining meaningful change. In order to crudely test this, the Reliable Change Index (RCI) was calculated using the method described by Jacobson and Truax [37], dividing the difference between the pre-treatment and post-treatment scores by the standard error of the difference between the two scores. This is a recommended method to ensure that the difference in scores is not simply due to measurement error [38].

## 3. Results

### 3.1. Recruitment and Retention

**Figure 2:**
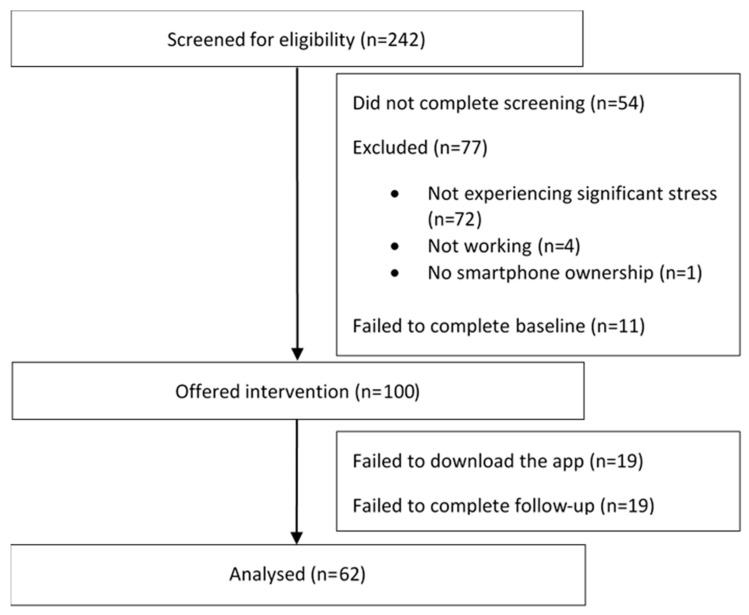
Flow of participants through the study.

There were more than 400 click-throughs from the social media advertisements, with 242 individuals consenting to screening over a 4-day period. Of these, 54 individuals failed to complete the screening and 77 did not meet the study inclusion criteria or were screened out. Subsequently, 11 eligible individuals did not complete baseline and did not receive the app. All 100 baseline completers were offered the app, with 19 failing to download the app. Those who downloaded the app were significantly more likely to have recently sought mental health support than those who did not (χ^2^_1_ = 4.42; *p* = 0.036); there were no other significant differences between these two groups. Of the 81 app users, 23.4% failed to complete the follow-up questionnaire, resulting in complete data on 62 users. Follow-up completers were found to engage in significantly more of the intervention (t_78_ = −4.73; *p* < 0.001). See Figure 2 (Consolidated Standards of Reporting Trials diagram) for details of participant flow through the study.

### 3.2. Sample Characteristics

Descriptive characteristics of the sample are reported in Table 1. The sample (participants who both completed baseline and downloaded the app; N = 81) was predominantly female (67.9%), with a mean age of 38.96 (SD = 10.34). Participants came from a wide range of industries with the majority working in the service sector, especially healthcare and social assistance (29.6%). Over 80% reported an episode of poor mental health lasting at least one month in the prior two years, with half (46.9%) having sought some form of mental health support in the previous month (e.g., from a GP, mental health professional, telephone or online support services, family, partner, or friends), and 35.8% were currently using medication for a mental health issue.

### 3.3. Symptom Change

On average, participants reported feeling moderately depressed at baseline (see Table 2), with a third (29.6%) of the overall sample meeting PHQ-9 algorithm criteria for likely major depression. At 5-week follow-up, participants reported significant reductions in depressive symptoms (t_61_ = 6.35; *p* < 0.001) and anxiety symptoms (t_60_ = 7.35; *p* < 0.001) (Table 2). Additionally, McNemar’s test showed that participants had significantly reduced rates of PHQ-9 caseness depression at follow-up compared to baseline (24% vs. 6%, *p* = 0.012). Significant improvements were also found in wellbeing (t_60_ = −5.64; *p* < 0.001), resilience (t_60_ = −3.89; *p* < 0.001), stress (t_61_ = 11.18; *p* < 0.001), and self-reported alcohol use (t_60_ = 3.40; *p* = 0.001). These changes all met RCI thresholds for reliable change. No change was found for past month self-reported work performance, absenteeism, or exercise. The results were largely consistent across male and female participants, with the exception of resilience and alcohol use, which did not show a significant change for males from baseline to follow-up. However, these calculations were underpowered due to the small number of male participants in the study.

Further analyses examined symptom change scores across participants meeting PHQ-9 algorithm criteria for MDD (case) versus not meeting criteria (non-case) at baseline assessment. Significant reductions in depressive symptoms from baseline to follow-up were seen both for cases (t_14_ = 6.18; *p* < 001) and non-cases (t_46_ = 4.37; *p* < 001). Similarly, for secondary outcomes that showed significant change overall, these changes remained significant for both MDD cases and non-cases.

Additional analyses were conducted to determine whether improvement in symptomatology and functioning was related to app usage. The results showed a significant association between change in depressive symptoms and number of challenge days completed (F_1,60_ = 6.28, *p* = 0.015; R^2^ = 0.10). Similarly, there were significant associations between challenge completion and change in wellbeing (F_1,59_ = 6.49, *p* = 0.013; R^2^ = 0.10), resilience (F_1,59_ = 7.53, *p* = 0.008; R^2^ = 0.11), stress (F_1,60_ = 14.67, *p* < 0.001; R^2^ = 0.20), and work performance (F_1,59_ = 4.715, *p* = 0.034; R^2^ = 0.07). No other comparisons reached significance.

### 3.4. App Usage

On average, users completed over a third of the challenge days (M = 12.86; SD = 11.82). Specifically, 86.4% completed at least one challenge day, 55.6% completed at least seven, and 20.9% completed all 30 days. Of those who failed to complete the 30-day challenge, other elements of the app may have been utilised (e.g., toolbox, support service information, mood/sleep/activity monitoring, or risk calculator).

### 3.5. Usability and Engagement

Most users found the design of the app at least somewhat interesting/engaging (84.7%), and the content at least somewhat interesting/engaging (89.8%). In addition, 93.2% found it easy to use.

When comparing participant feedback on the usefulness of specific therapeutic elements within the challenge, there was a fairly even spread between the cognitive elements (e.g., cognitive challenging, grounding, problem solving), behavioural elements (value-driven activity planning and goal-setting), and mindfulness components (preferred by 33.9%, 30.5%, and 25.4% of participants respectively). The psychoeducational videos were less popular (preferred by 8.5% of participants). Of those who completed follow-up, 47.5% claimed to have finished the challenge or were still completing it, while the most popular reasons for non-completion were a lack of time (23.7%) and issues of lost interest or motivation (10.2%).

### 3.6. Feasibility and Acceptability

The app was well-received by participants, with 83.0% claiming it had at least moderately improved their mental fitness, and only 6.8% claiming that they felt no improvement. The majority (83.0%) claimed they understood the app content either very well or completely, while 93.2% stated that they would recommend the app to others. Overall, 72.9% of participants rated the app highly or very highly.

### 3.7. App Feedback 

Open feedback on the app was generally positive: “I really enjoyed using the app, it helped me to think things thru more! Thank you”. Additional reminders and adjunct components were also suggested: “I found the content of the app incredibly useful, the videos and how everything was worded was very easy to understand, I definitely learnt a few things. I think I will have trouble using the techniques in real life without someone face-to-face checking in and reminding me to do things”. Most criticisms related to confusion around navigation within the app, e.g., “I didn’t understand where to find different things in the app… Overall, it’s a great concept and something with the potential to be of great use to people. But I got confused…” As such, suggestions for improvement were largely focused on elements of user interface and user experience design to improve clarity in this regard: “Improve UX flows please, finding stuff is hard. Larger fonts and tap targets, clearer labels would be good”. Other suggestions referred to specific pages and areas within the app that could be improved: “Wasn’t that drawn to map and wheel house—the look and feel may be a little basic and not appealing”; “whole home page needs a refresh”.

## 4. Discussion

This pilot study aimed to investigate the usability, feasibility, acceptability, and preliminary effects of Anchored, an app-based mental health intervention. Based on the findings, there is promising evidence that Anchored may improve the mental health and wellbeing of stressed workers. The app was also found to have satisfactory levels of engagement, usability, feasibility, and acceptability, indicating that it is suitable for investigation in a controlled trial with a large population sample.

The observed shift in mean depression scores from the moderate level to the mild level represents not only a significant reduction in depressive symptoms from pre- to post-intervention, but also a clinically significant change according to Jacobson and Truax [37] whereby post-treatment scores fall within two standard deviations of the normal population mean score [39]. Although the 4-point mean change in PHQ-9 scores was just short of the 5-point recommendation for clinical importance [40], this recommendation is based on a clinically unwell population. It is necessary to highlight that the present study did not specifically target such a group, with only a quarter of participants meeting PHQ-9 algorithm criteria for MDD at baseline and a third using medication for a mental health issue. As such, the significant reduction in depressive symptoms within this heterogeneous group suggests that Anchored may have public health benefits, particularly as a selective prevention intervention for at-risk individuals such as those experiencing elevated workplace stress.

While the PHQ-9 is not a diagnostic tool, use of the PHQ-9 diagnostic algorithm allows self-reported symptoms to be mapped against the DSM-IV criteria for major depressive disorder, to identify likely cases [29]. For participants who were defined as having likely MDD at baseline, the mean change in PHQ-9 score from pre- to post-intervention was over 7 points, reaching the threshold of clinical importance for individual change [40]. Crucially, based on the PHQ-9 algorithm, this translated to a significant reduction in cases of depression.

Further, when comparing likely baseline MDD cases and non-cases, significant positive changes were seen across both groups, for depressive symptoms along with secondary outcomes including anxiety, stress, wellbeing, and resilience. With the caveat that this study did not involve face-to-face clinical assessment, our results provide preliminary evidence that Anchored may have utility regardless of initial depressive symptom levels. These findings warrant further investigation into the potential application of Anchored both as a selective and indicated prevention/early intervention tool.

Importantly, despite only a small scale yielding a very limited number of discreet scores, significant change in participant stress was evident. This is particularly encouraging, as longitudinal data shows that high work stress may lead to depression and anxiety, even in individuals with no previous history of disorder [15]. It follows that a reduction in perceived work stress may reduce the risk of future mental ill health, with concomitant benefits for individual wellbeing and work productivity. Inclusion of a more sensitive measure of stress will enable further investigation into the app’s potentially beneficial role in stress reduction.

No significant change was seen in work performance or absenteeism, although encouragingly, more intervention completion was associated with significant improvements. However, this pilot study included a 5-week follow-up assessment only, and it is uncertain whether changes in work performance could be detected within this short follow-up period. There is a need for long-term follow-up assessments to capture potential flow-on effects from the observed improvements in depression and anxiety symptoms, stress, and wellbeing. This is suggested by findings from the HeadGear randomised controlled trial, where no change was found in work productivity immediately post-intervention, whereas significant improvement was observed at both 3- and 12-month follow-up [20].

The significant reductions seen in depressive symptoms, anxiety symptoms, stress, and alcohol use, along with significantly increased wellbeing and resilience, are overall very encouraging. These results are bolstered by the finding that more intervention completion was associated with greater improvements across most of the significant outcomes (with the exception of alcohol use), which is consistent with evidence from systematic reviews showing that the level of program or module completion is positively correlated with outcomes in eHealth interventions for depression and anxiety [41]. This does not necessarily mean that the entire intervention must be completed to achieve positive results [42], for example some users may get what they need from the intervention within a week, and others may require the full 30-day challenge. In terms of intervention adherence, users completed on average almost 13 out of 30 Anchored challenge days, which compares favourably with previous studies [18]. Further investigation is required to determine the minimum intervention dosage required to achieve positive change, in order to operationalise “adherence” to the Anchored intervention [43]. In addition, due to the small sample size in this study, we were unable to conduct dose-response calculations to determine the optimal level of intervention required. These questions will be explored in greater depth when trialling this app with a larger population.

User engagement, while related to intervention adherence, is a distinct concept that is just as important to consider [44]. It may be that app engagement is a more critical factor for achieving the intervention effect than a simple metric of app usage [45,46]. User feedback regarding Anchored content and interest/engagement was generally positive, with lack of time by far the most prevalent reason for non-completion, while lost interest or motivation was a factor for only 10% of those who did not complete the 30-day challenge. Of course, this does not include those lost to follow-up. In any case, based on the pilot user feedback it will be possible to make qualitative improvements to the app in several areas to increase usability and engagement, and therefore increase the likelihood of user retention, prior to testing in a larger-scale RCT. Participants in this study identified issues particularly around app navigation and user interface, and also provided suggestions for improving usability, for example via larger fonts and clearer labels. This qualitative feedback will inform further refinements to the Anchored app in consultation with user experience specialists, to modify navigation flows and improve accessibility. Proposed design changes include: (1) improved navigation via refined home screen emphasising key app elements and challenge flow; (2) more visually engaging control centre and toolbox sections with an additional “favourite tools” feature; and (3) increased accessibility via clearer instructions and larger fonts and icons throughout the app. The study also highlighted the need for more nuanced app usage data to further explore use patterns and the uptake of specific elements. 

There are several limitations to this study. Firstly, and most importantly, the uncontrolled nature of this pilot study limits the findings as we were unable to control for factors such as spontaneous symptom improvement, regression to the mean, the effect of medication, use, or concurrent psychological treatment. This issue will be addressed via future testing of the app in an RCT with both an intervention and comparison group. Secondly, while Anchored was designed to prevent new onset depression, unfortunately power and sample size constraints make this difficult in a pilot study. In attempting to test the app in a real-world setting, we applied no restriction on baseline depression severity, and used symptom change as a proxy for impact. Although we explored outcomes separately in those meeting and not meeting the PHQ-9′s threshold for probable depression, the impact of the app in terms of “true” prevention (i.e., using a strictly non-clinical sample) requires more rigorous testing. Thirdly, as recruitment was conducted via social media rather than directly through organisations/workplaces, this may have drawn a biased sample that is not representative of the general workforce (for example, these participants may be more comfortable using digital technology). However, the online recruitment method allowed for sampling from a diverse range of industries, which may not have been possible otherwise. Finally, participant attrition may have influenced the results, both in terms of participants who completed the baseline assessment but did not download the app, and those who utilised the app but did not complete the follow-up assessment. There were no significant differences in demographics or baseline symptom levels between downloaders and non-downloaders, although non-downloaders were less likely to have recently sought help for mental health issues, indicating that this group may have been in a precontemplation stage of change [47]. Regarding those who used the app but did not complete the follow-up assessment, in-app data showed that these participants completed less of the intervention, suggesting they may have been less engaged overall. This was, however, a relatively small portion of the overall sample.

## 5. Conclusions

This pilot study indicates that the Anchored app was well-received and displayed satisfactory levels of usability, acceptability, feasibility, and engagement. Importantly, the study indicated strong preliminary evidence that the app may reduce depressive symptoms and improve a range of other mental health outcomes in the working population. Comparison of the Anchored app with a control condition in a larger randomised controlled trial will help to address the limitations of the present study and further elucidate these promising findings.

## Figures and Tables

**Table 1:** Sample characteristics.

	N = 81 (%)
**Mean age (*SD*)**	38.96 (10.34)
**Gender**	
Female	55 (67.9)
**Industry**	
Healthcare and social assistance	24 (29.6)
Administration and support services	9 (11.1)
Education and training	8 (9.9)
Professional, scientific and technical services	7 (8.6)
Wholesale and retail trade	6 (7.4)
Accommodation and food services	5 (6.2)
Information media and telecommunications	5 (6.2)
Construction; electricity, gas, water, and waste services	5 (6.2)
Other services (e.g., financial; arts and recreation; real estate; manufacturing; agriculture, forestry, and fishing)	12 (14.8)
**Education**	
Below year 12 certificate	3 (3.7)
Year 12 certificate	12 (14.8)
Trade or other certificate	13 (16.0)
Diploma	11 (13.6)
University degree	42 (51.9)
**Previous episode of poor mental health (last 2 years)**	71 (87.7)
**Current medication for mental health issue**	29 (35.8)

**Table 2:** Change in outcome scores over time.

	Pre-Trial Mean (SD)	Post-Trial Mean (SD)	t (df)	*p*	Effect Size (95% CI)	Standardised Mean Difference	Reliable Change Index
PHQ-9 (Depression)	11.27 (5.24)	7.29 (4.54)	6.35 (61)	<0.001	0.76 (0.42, 1.10)	0.76	6.39
WHO-5 (Wellbeing)	7.75 (4.35)	11.46 (5.40)	−5.64 (60)	<0.001	−0.85 (−1.20, −0.51)	0.76	5.65
GAD-7 (Anxiety)	9.64 (4.23)	5.75 (3.26)	7.35 (60)	<0.001	0.92 (0.57, 1.27)	1.03	7.37
BRS (Resilience)	2.83 (0.69)	3.16 (0.76)	−3.89 (60)	<0.001	−0.48 (−0.81, −0.14)	0.46	3.93
Alcohol use	4.07 (1.56)	3.80 (1.56)	3.40 (60)	0.001	0.17 (−0.16, 0.50)	0.17	3.51
Exercise	3.64 (1.33)	3.51 (1.55)	1.11 (60)	0.271	0.10 (−0.23, 0.43)	0.09	1.10
SISQ (Stress)	4.23 (0.42)	3.08 (0.78)	11.18 (61)	<0.001	2.74 (2.28, 3.20)	1.92	11.27
Absenteeism	1.98 (4.17)	1.72 (5.07)	−0.18 (60)	0.861	0.06 (−0.27,0.39)	0.26	0.58
Work performance	0.61 (0.14)	0.64 (0.17)	−0.97 (60)	0.338	−0.21 (−0.55, 0.12)	0.19	1.25

PHQ-9: Patient Health Questionnaire-9. WHO-5: 5-item World Health Organisation Well-Being Index. GAD-7: Generalized Anxiety Disorder-7. BRS: Brief Resilience Scale. SISQ: Single-item Stress Question.
